# Evidence for Site-Specific Occupancy of the Mitochondrial Genome by Nuclear Transcription Factors

**DOI:** 10.1371/journal.pone.0084713

**Published:** 2014-01-20

**Authors:** Georgi K. Marinov, Yun E. Wang, David Chan, Barbara J. Wold

**Affiliations:** 1 Division of Biology, California Institute of Technology, Pasadena, California, United States of America; 2 Howard Hughes Medical Institute, Pasadena, California, United States of America; Univeristy of California Riverside, United States of America

## Abstract

Mitochondria contain their own circular genome, with mitochondria-specific transcription and replication systems and corresponding regulatory proteins. All of these proteins are encoded in the nuclear genome and are post-translationally imported into mitochondria. In addition, several nuclear transcription factors have been reported to act in mitochondria, but there has been no comprehensive mapping of their occupancy patterns and it is not clear how many other factors may also be found in mitochondria. Here we address these questions by using ChIP-seq data from the ENCODE, mouseENCODE and modENCODE consortia for 151 human, 31 mouse and 35 *C. elegans* factors. We identified 8 human and 3 mouse transcription factors with strong localized enrichment over the mitochondrial genome that was usually associated with the corresponding recognition sequence motif. Notably, these sites of occupancy are often the sites with highest ChIP-seq signal intensity within both the nuclear and mitochondrial genomes and are thus best explained as true binding events to mitochondrial DNA, which exist in high copy number in each cell. We corroborated these findings by immunocytochemical staining evidence for mitochondrial localization. However, we were unable to find clear evidence for mitochondrial binding in ENCODE and other publicly available ChIP-seq data for most factors previously reported to localize there. As the first global analysis of nuclear transcription factors binding in mitochondria, this work opens the door to future studies that probe the functional significance of the phenomenon.

## Introduction

Mitochondria are the primary site of ATP production through oxidative phosphorylation and are therefore critical to eukaryotic cells. It is widely accepted that they arose as the result of an endosymbiotic event [63] between the ancestor of modern eukaryotes and a member of the *α*-proteobacteria clade [82]. Reflective of the organelle's prokaryotic ancestry, mitochondria retain their own reduced circular genome [55], although its size has been greatly reduced in many eukaryotes through transfer of genes to the eukaryotic nucleus. After transcription and translation of nuclear components of the separate mitochondrial transcription, replication and regulatory machineries, a number of which retain evidence of their prokaryotic origin [74], the protein products are then imported back into the mitochondria to modulate organellar function.

The mitochondrial genome in mammals encodes 13 proteins, all of which are components of the electron transport chain, as well as 22 tRNAs and two rRNAs [3], [5]. Mitochondrial DNA (mtDNA) is organized in cells as macromolecular DNA-protein complexes called nucleoids. Mitochondrial genes are densely packed along the genome with the notable exception of the non-coding displacement loop (D-loop) regulatory region [66], which is located within the non-coding region (NCR). Transcription initiates in the D-loop, is carried out by the mitochondrial-specific RNA polymerase POLRMT, and results in long polycistronic transcripts from each strand (called the Heavy- or H-strand and the Light- or L-strand), from the light strand promoter (LSP) and two Heavy strand promoters (HSP1 and HSP2) [9], [52]. In addition, the transcription factors mtTFA/TFAM [27], [28] and mtTFB2/TFB2M as well as the methyltransferase mtTFB1/TFB1M [26], [29], [49] are required for initiation and regulation of transcription [69]. Unlike many of the proteins involved in regulation of the mitochondrial genome, these transcription factors are generally accepted as not being of prokaryotic origin. Instead, they are genes of eukaryotic ancestry, appropriated for their function through co-evolution of the organellar and cellular genomes and imported into mitochondria to regulate mtDNA transcription.

In addition to these well-characterized regulators of mitochondrial transcription, multiple reports have suggested that transcription factors that typically act in the nucleus might also have regulatory functions in mitochondrial transcription [44], [73]. The glucocorticoid receptor (GR) was the first such factor reported to localize to mitochondria and to interact with mtDNA [18], [19], [40], [59]. A 43 kDa isoform of the thyroid hormone T_3_ receptor T_3_R*α*1 called p43 has been found to directly control mitochondrial transcription [11], [24], [25], [81]. Cyclic-AMP Response element Binding protein (CREB) has been shown to localize to mitochondria and suggested to bind to the D-loop [8], [17], [43], [62]. The tumor suppressor transcription factor p53 has been implicated in mtDNA repair and regulation of gene expression through interactions with TFAM [1], [34], [47], [48], [83]. It has also been proposed to play a proapoptotic role through association with the outer mitochondrial membrane [76]. A similar role has been also ascribed to the IRF3 transcription factor [12], [46]. The mitochondrial localization of the estrogen receptor (ER) is also well established, for both its ER*α* and ER*β* isoforms, and it too has been suggested to bind to the D-loop [13], [51]. NF*κ*B and I*κ*B*α* have been found in mitochondria and have been proposed to regulate mitochondrial gene expression [16], [36]. The AP-1 and PPAR*γ*2 transcription factors have been proposed to localize to mitochondria and bind to the genome. [10], [57], [58] and the MEF2D transcription factor was found to regulate the expression of the ND6 gene by binding to a consensus sequence recognition motif within it [67]. Finally, the presence of STAT3 in mitochondria has been found to be important for the function of the electron transport chains and also to be necessary for TNF-induced necroptosis [32], [68], [71], [72], [79], although direct mtDNA binding has not been established. Mitochondrial localization has also been reported for STAT1 and STAT5 [6], [14].

However, direct *in vivo* chromatin immunoprecipitation evidence for the binding of these factors to mtDNA exists only for CREB [43], p53 [1] and MEF2D [67], and with the exception of MEF2D characterization is limited to the D-loop region. No prior studies have assayed transcription factor occupancy across the entire mitochondrial genome in vivo with modern high resolution techniques such as ChIP-seq (Chromatin Immunoprecipitation coupled with deep sequencing, [35]). As a result, the precise nature, and in many instances the existence, of the proposed binding events remains unknown. The limited sampling of transcription factors in previous studies also leaves uncertain how common or rare localization to mitochondria and binding to mtDNA is for nuclear transcription factors in general.

Here we survey the large compendium of ChIP-seq and other functional genomic data made publicly available by the ENCODE, mouseENCODE and modENCODE Consortia [22], [23], [30], [50], [54] to identify transcription factors that associate directly with mtDNA and to characterize the nature of these interactions. We identify eight human and three mouse transcription factors for which robust evidence of site-specific occupancy in the mitochondrial genome exists. These sites exhibit the strand asymmetry typical of nuclear transcription factor binding sites, usually contain the recognition motifs for the factors in question, and are typically the strongest (as measured by ChIP-seq signal strength) binding sites found in both the nuclear and mitochondrial genome by a wide margin. Notably, these interactions are all found outside of the non-coding D-loop region. The D-loop region itself exhibits widespread sequencing read enrichment for dozens of transcription factors. However, it does not show the aforementioned feature characteristics of true binding events. Though not observed in control datasets generated from sonicated input DNA, the high ChIP-seq signal over the D-loop is frequently seen in control datasets generated using mock immunoprecipitation, suggesting that it is likely to represent an experimental artifact. Examination of available ChIP-seq data for the transcription factors previously proposed to play a role in mitochondria (GR, ER*α*, CREB, STAT3, p53) revealed no robust binding sites except for enrichment in the D-loop. Resolving the functional significance of the identified occupancy sites in future studies should provide exciting insights into the biology of both mitochondrial and nuclear transcriptional regulation.

## Results

**Figure 1:**
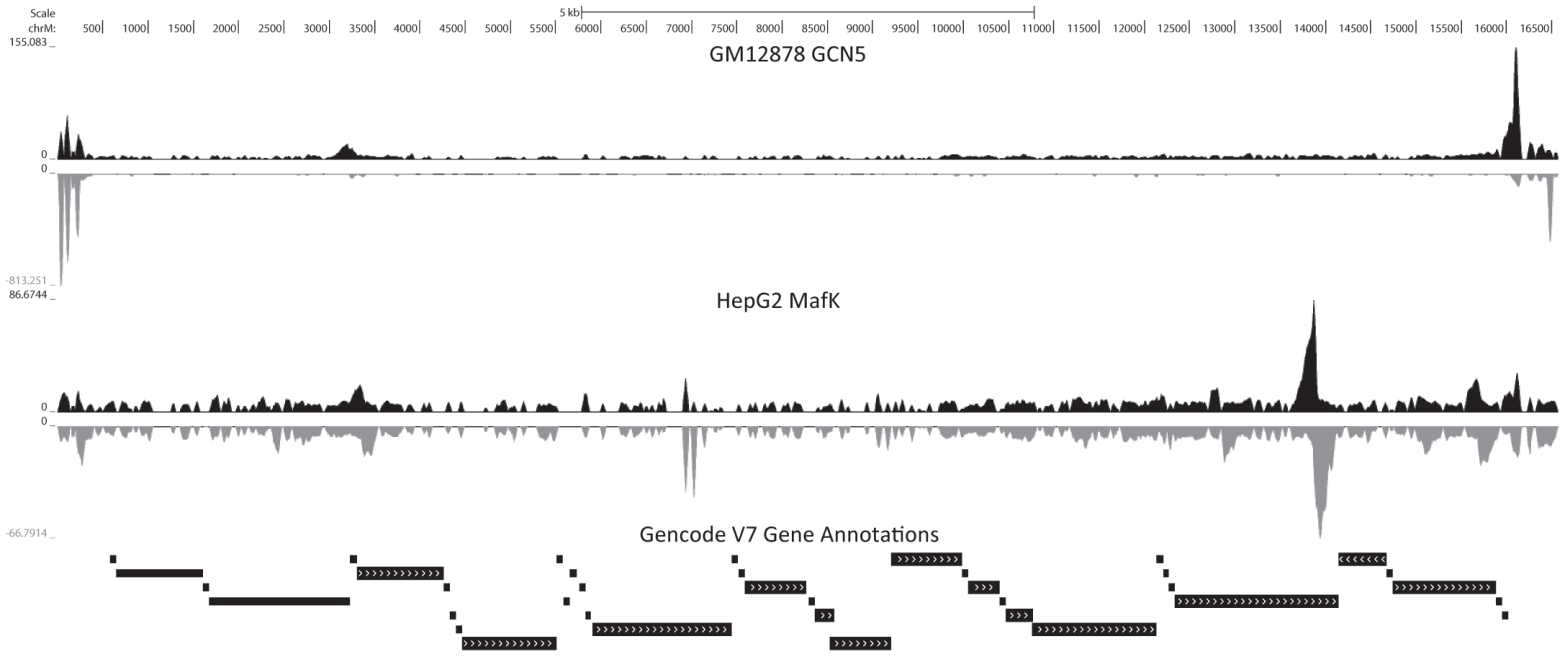
Representative USCS Genome Browser snapshots of nuclear transcription factor ChIP-seq datasets exhibiting strong enrichment in the mitochondrial genome. (A) GM12878 GCN5 shows high signal intensity in the D-loop (the region between coordinates 16030 and 580, i.e. the non-coding regions on the left and right ends of the snapshot) representative of the D-loop enrichment observed for a large number of transcription factors (B) In contrast, a large MafK peak is observed in a coding region outside of the D-loop in HepG2 cells. Upper track (black) shows reads aligning to the forward strand, lower track (gray) shows read aligning to the reverse strand.

In the course of a study of TFAM occupancy in the mitochondrial and nuclear genomes [78], we noticed that a number of nuclear transcription factors exhibit localized enrichment in certain areas of the mitochondrial genome in ChIP-seq data (Figure 1). These events could be divided in two classes: high ChIP-seq signal over the NCR, and localized high read density over regions outside of it. Given prior reports suggesting that nuclear transcription factors might act in mitochondria, this prompted us to determine the general prevalence of the phenomenon among transcription factors and investigate evidence of occupancy in detail, as the power and resolution of ChIP-seq have not previously been brought to bear on this somewhat mysterious phenomenon. We took advantage of the wide compendium of human, mouse, fly and worm functional genomics data generated by the ENCODE [22], [23], mouseENCODE [54] and modENCODE [30], [50] consortia.

### Identifying transcription factor binding events in the mitochondrial genome

We downloaded publicly available (as of February 2012) ENCODE and mouseENCODE ChIP-seq and control data from the UCSC Genome Browser and modENCODE data from ftp://ftp.modencode.org, including ChIP-seq data for 151 transcription factors in human cell lines [77], 31 in mouse and 35 in *C.elegans* (see discussion on *D. melanogaster* below). We also downloaded DNase hypersensitvity (both DNase-seq [75] and Digital Genomic Footprinting (DGF) [56]), FAIRE-seq (Formaldehyde Assisted Isolation of Regulatory Elements) [70] and MNase-seq data as these datasets provide valuable orthogonal information about potentially artifactual patterns of read enrichment over the mitochondrial genome.

**Figure 2:**
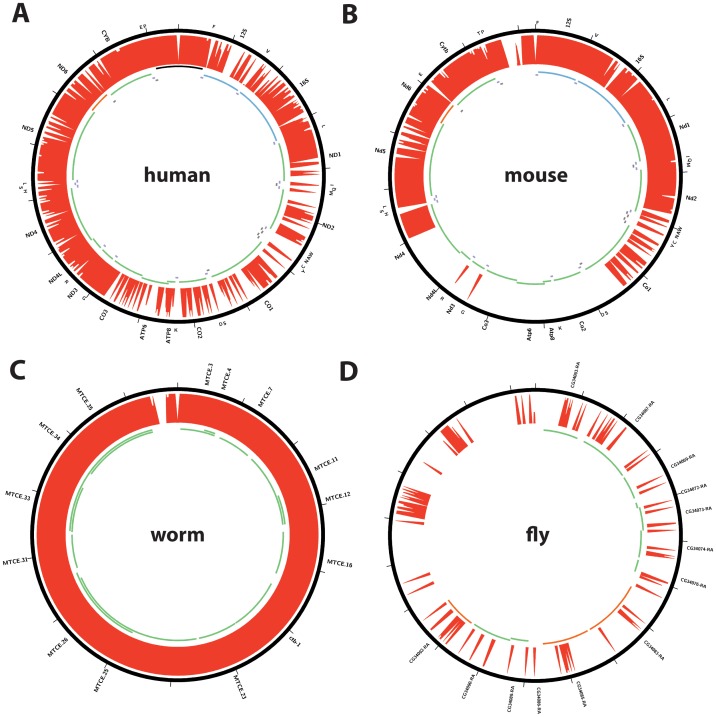
Unique mappability of the mitochondrial genome (chrM) in ENCODE and modENCODE species. (A) human; (B) mouse; (C) *C. elegans*; (D) *D. melanogaster*. The 36 bp mappability track (see Methods for details) is shown. The annotated protein coding and rRNA and tRNA genes are shown in the inner circles as follows: forward-strand genes are shown as green lines, while reverse-strand genes are shown as red lines, with the exception of mouse and human rRNA and tRNAs (blue). The D-loop region in human is shown in black. Gene annotations were obtained from ENSEMBL (version 66). Plots were generated using Circos version 0.60 [41].

It is well known that the nuclear genome contains partial copies of the mitochondrial genome (**NU**clear **M**i**T**ochondrial sequences or NUMTs) [20], [33]. Depending on their levels of divergence from the mitochondrial sequence, they can present an informatics challenge for distinguishing binding events to the true mitochondrial genome from binding events to NUMTs. For this reason, we aligned reads simultaneously against the nuclear and mitochondrial genomes. We then retained only reads that map uniquely, and with no mismatches, relative to the reference for further analysis (see Methods for details). As a consequence this stringent mapping strategy, regions of the mitochondrial genome that are also present as perfectly identical copies in the nuclear genome are “invisible” to our analysis; this was a necessary compromise in order to focus only on a maximally stringent set of putative mitochondrial binding events. However, before proceeding, we examined how widely affected the mitochondrial genome is by this treatment in the four relevant species by generating mappability tracks (shown in Figure 2). The human mitochondrial genome contains numerous small islands of unmappable sequence, particularly concentrated between the ND1 and CO3 genes, but it displays no large completely unmappable segments (Figure 2A). The mouse genome contains a large unmappable stretch between the CO1 and ND4 genes (Figure 2B). The *C. elegans* mitochondrial genome is almost completely uniquely mappable (Figure 2C). In contrast, the *D. melanogaster* genome is almost completely unmappable, indicating the presence of very recent insertions into the nuclear genome with high sequence similarity. We therefore excluded fly datasets from further analysis and focused on human, mouse and worm data.

**Figure 3:**
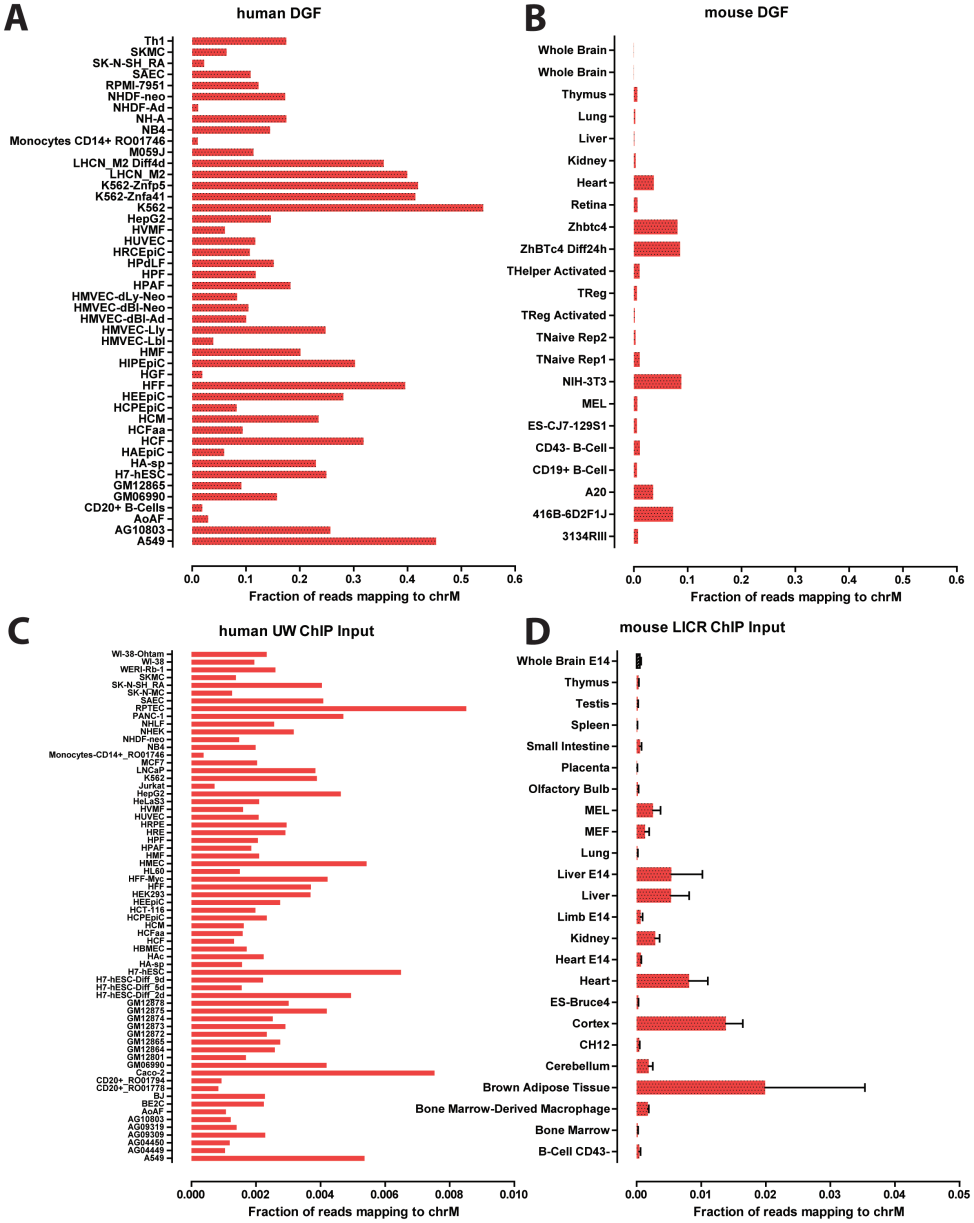
Variation in mitochondrial DNA copy number in cell lines and tissues. The fraction of reads mapping to the mitochondrial genome (chrM) is shown. (A,B) UW human (A) and mouse (B) UW ENCODE digital genomic footprinting (DGF) data; (C) UW human ChIP input datasets; (D) LICR mouse ChIP input datasets. “UW” and “LICR” refers to the ENCODE production groups that generated the data. Inputs from the UW and LICR groups were chosen because they are the largest ENCODE sets in terms of number of cell lines/tissues assayed by the same production groups, thus avoiding possible variation between different laboratories. A general positive correlation between the expected metabolic demand of the tissue type and the relative amount of reads mapping to chrM is observed.

Mammalian cells typically contain hundreds to thousands of copies of mtDNA, with the precise number varying depending on the metabolic needs of the particular cell type [7], [64], [80]. This variation is relevant to our analysis because the relative read density over the mitochondrial genome is expected to scale with the mtDNA∶nuclear DNA ratio for a given cell. Thus, cell types with very high mtDNA copy number are expected to display correspondingly elevated background read density over the mitochondrial genome. Several types of ENCODE data provide a rough proxy for the relative mitochondrial genome copy number per cell. In particular, the fraction of reads originating from the mitochondrial genome in DNase hypersensitivity and ChIP control datasets is expected to scale accordingly. We examined the distribution of this fraction in ENCODE and mouseENCODE DGF datasets and observed very large differences between different cell lines and tissues (Figure 3). For example, about half of reads in K562 DGF data originated from mitochondria, while the fraction was less than 2% in CD20+ B-cells (Figure 3A). Notably, these differences are in many cases (though not always) consistent with what is known about the cell lines, with certain cancer cell lines (such as K562 and A549) and muscle cells (LHCN) showing the largest number of mitochondrial reads, while primary cells with small volumes of cytoplasm such as B-cells showed the least.

Mouse DGF data was available mostly for tissues, and the fraction of mitochondrial reads in these was much smaller compared to both the human cell lines and the few mouse cell lines assayed (Figure 3B). This is consistent with a significant proportion of cells in tissues being in a less active metabolic state than cell lines in culture. Still, we observed expected differences between tissues. For example, one of the tissues that was most enriched for reads mapping to the mitochondrial genome was the heart. We observed similarly large differences in ChIP control datasets (Figure 3CD), although the absolute number of reads was much lower than it was in DGF data. Again, the mouse tissues with the highest number of mitochondrial reads were the more metabolically active ones, such as brown adipose tissue, cortex, and heart.

These large differences in background read coverage between different cells lines/tissues have two consequences for the analysis of putative transcription factor binding to the mitochondrial genome. First, peak calling algorithms usually used to identify transcription factor binding sites from ChIP-seq data may not work equally well in different cell lines due to the highly variable background read density. Second, these differences render comparing the strength of binding across cell lines difficult.

We therefore devised a normalization procedure (described in Methods) to convert read coverage to signal intensity z-scores reflecting how strongly regions of enrichment stand out compared to the average background read density along the mitochondrial genome for each dataset. We then used the maximum z-scores for each dataset to identify datasets with very strong such enrichment, which we then examined manually in detail.

### Nuclear transcription factor binding to the mitochondrial genome in human cell lines

**Figure 4:**
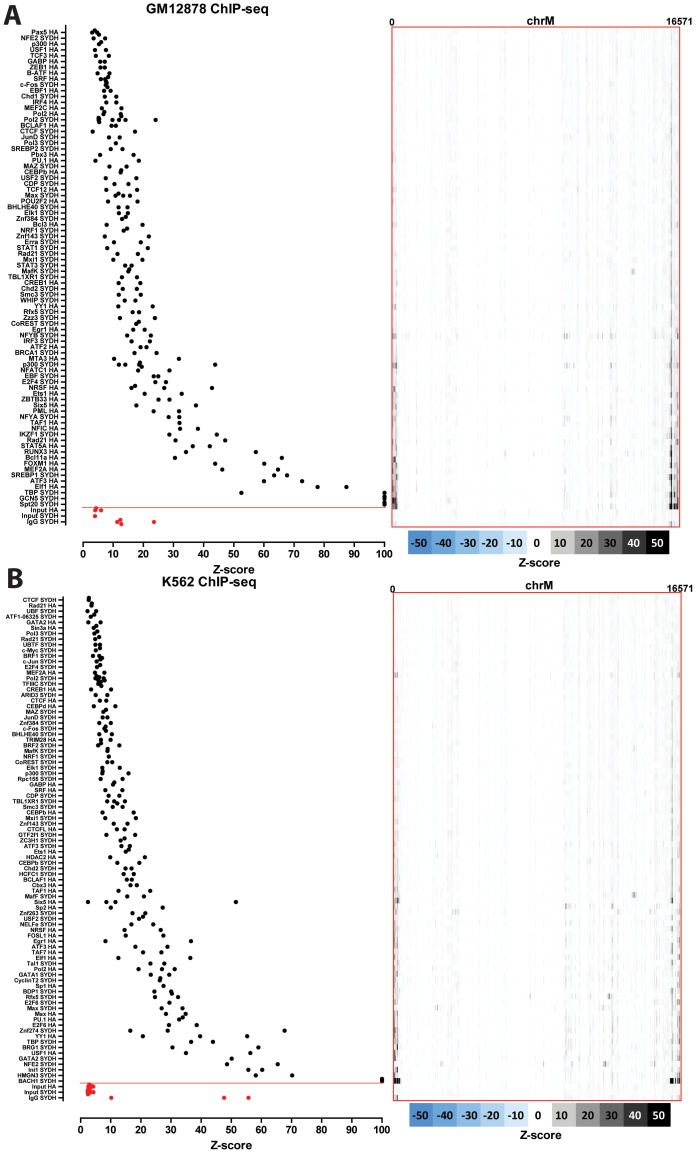
Signal distribution over the mitochondrial genome in human ChIP-seq datasets. The maximum z-score for each individual TF ChIP-seq replicate in each cell line is shown on the left (factors are sorted by average z-score, with control datasets always shown on the bottom in red, below the red horizontal line). The z-score profile along the mitochondrial chromosome for the replicate with the highest z-score is shown on the right. “SYDH” and “HA” refer to the ENCODE production groups which generated the data. Z-scores ≥100 are shown as equal to 100. (A) GM12878 cells; (B) K562 cells.

**Figure 5:**
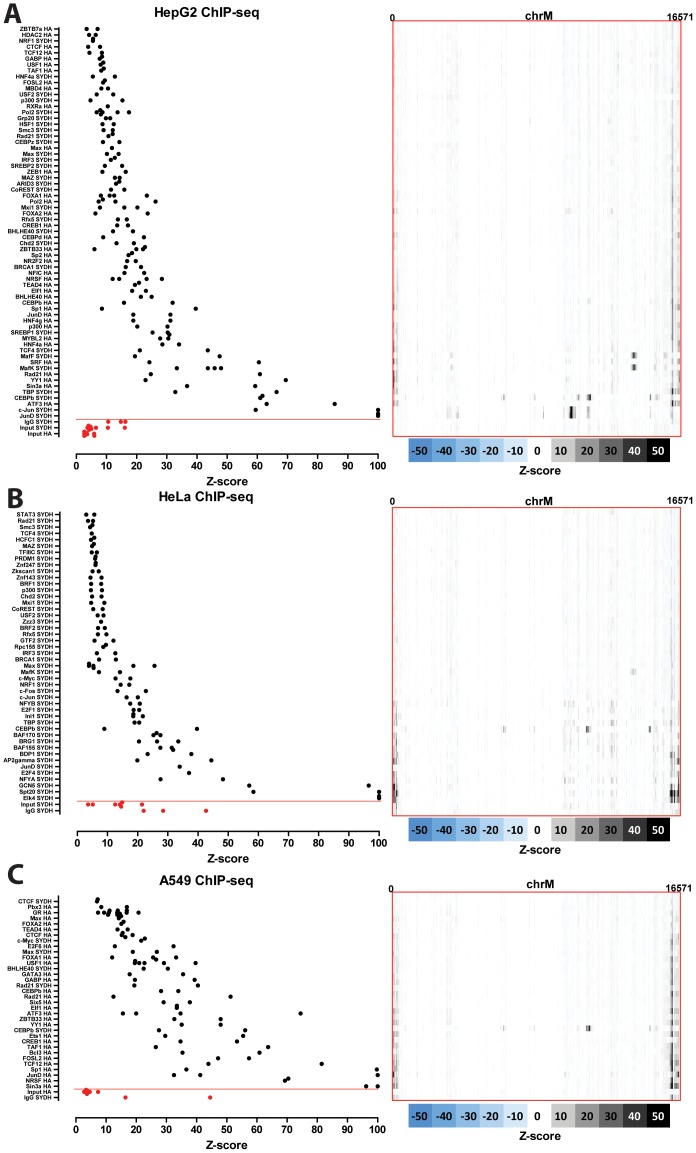
Signal distribution over the mitochondrial genome in human ChIP-seq datasets. The maximum z-score for each individual TF ChIP-seq replicate in each cell line is shown on the left (factors are sorted by average z-score, with control datasets always shown on the bottom in red, below the red horizontal line). The z-score profile along the mitochondrial chromosome for the replicate with the highest z-score is shown on the right. “SYDH” and “HA” refer to the ENCODE production groups which generated the data. Z-scores ≥100 are shown as equal to 100. (A) HepG2 cells; (B) HeLa cells; (C) A549 cells.

**Figure 6:**
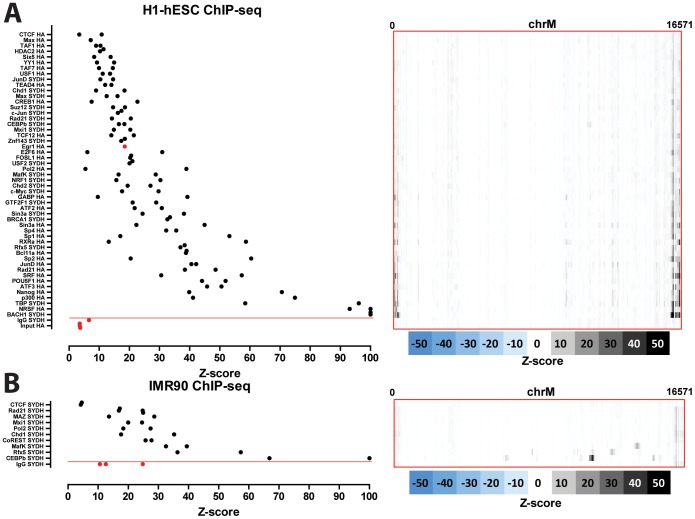
Signal distribution over the mitochondrial genome in human ChIP-seq datasets. The maximum z-score for each individual TF ChIP-seq replicate in each cell line is shown on the left (factors are sorted by average z-score, with control datasets always shown on the bottom in red, below the red horizontal line). The z-score profile along the mitochondrial chromosome for the replicate with the highest z-score is shown on the right. “SYDH” and “HA” refer to the ENCODE production groups which generated the data. Z-scores ≥100 are shown as equal to 100. (A) H1-hESC cells; (B) IMR90.

**Figure 7:**
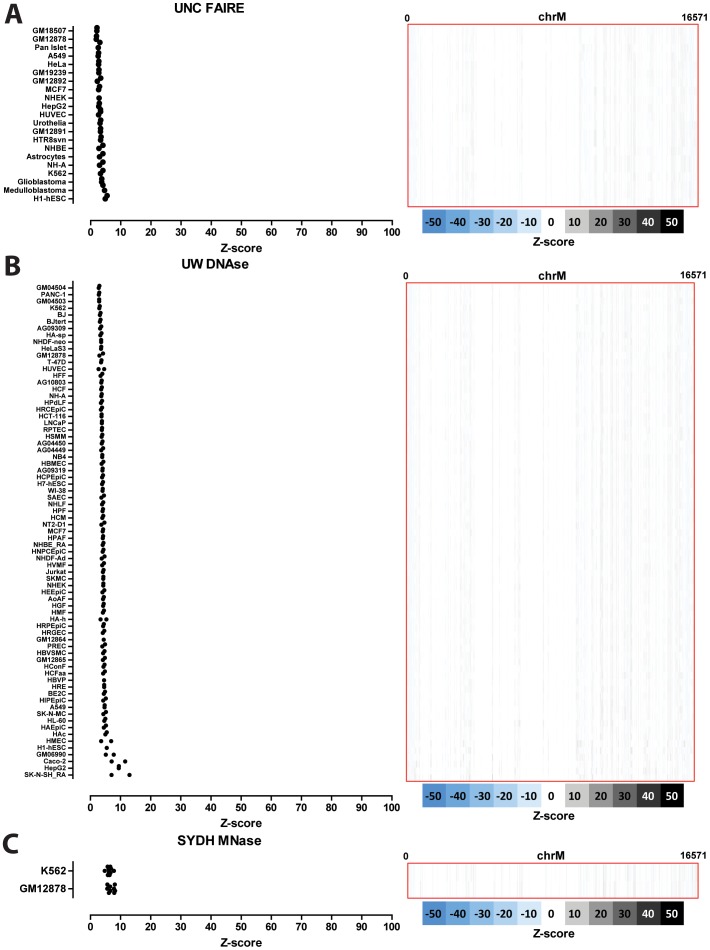
Signal distribution over the mitochondrial genome in human FAIRE-seq, DNAse-seq and MNAse-seq datasets. Shown is the maximum z-score for each individual replicate for each cell line (left) and the z-score profile along the mitochondrial chromosome for the replicate with the highest z-score (right). (A) FAIRE data; (B) DNAse data; (C) MNAse data. “UNC”, “UW” and “SYDH” refer to the ENCODE production groups which generated the data. Z-scores larger than 100 are shown as 100. No read enrichment over the D-loop is observed, suggesting that the D-loop signal found in TF ChIP-seq datasets is not due to sequencing biases but is a result of the immunoprecipitation process.

**Figure 8:**
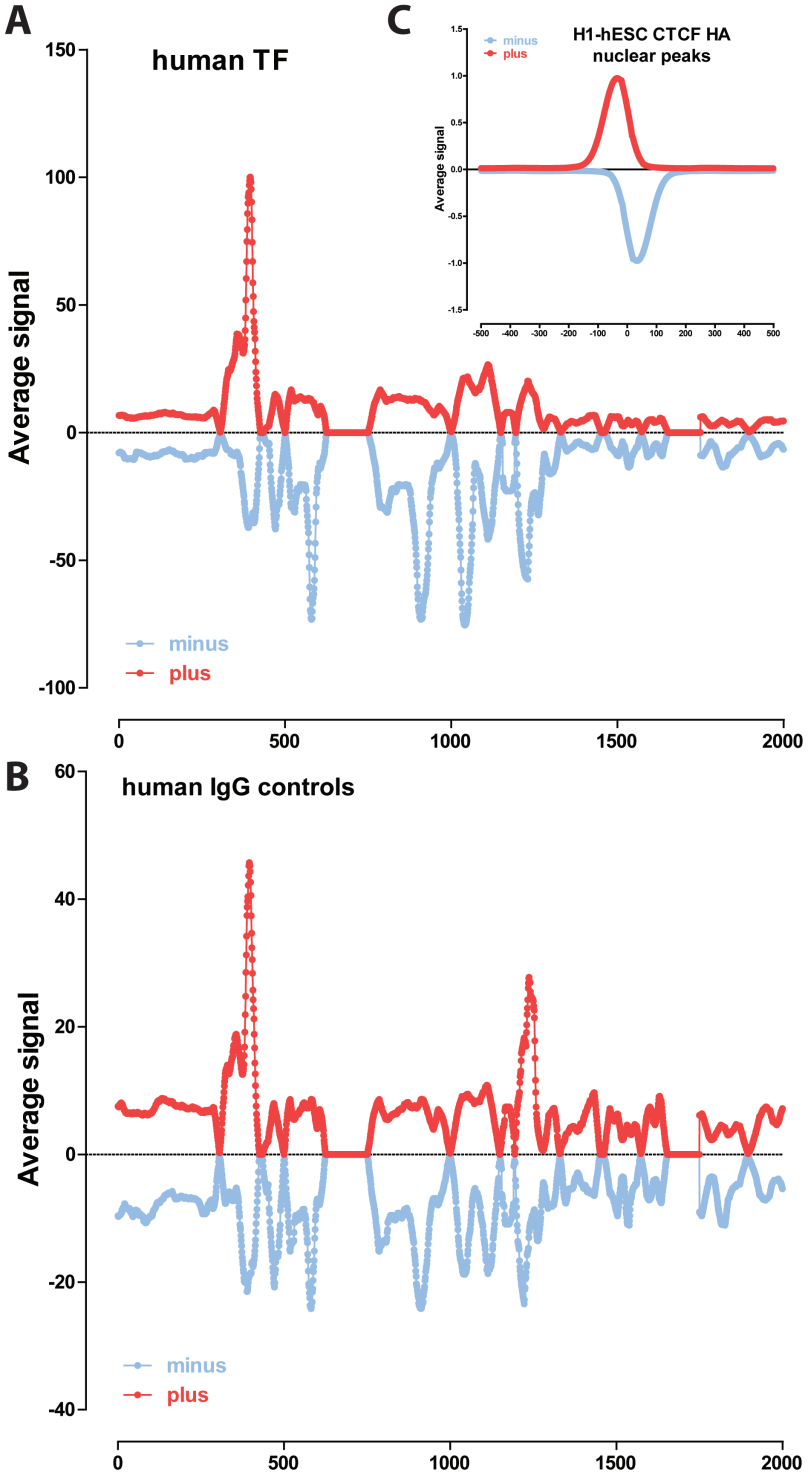
Combined signal distribution profile for the forward and reverse strand in the D-loop region. Shown is the average signal (in RPM) for each strand in human ChIP-seq datasets with z-scores ≥20 (A) and human IgG controls (B). Also shown for comparison is the plus and minus strand read distribution around nuclear CTCF binding sites in H1-hESC cells (C).

The distribution of read density z-scores for transcription factor ChIP-seq and control datasets in seven ENCODE human cell lines (GM1278, K562, HepG2, HeLa, H1-hESC, IMR90 and A549) is shown in Figures 4, 5 and 6. A wide range in the values of the maximum z-score is observed, from less than 5, to more than 100. Strikingly, most factors exhibit high read density in the NCR. One obvious explanation for this observation is that it represents an experimental artifact. This is likely, as the NCR contains the D-loop [66], the unique triple-strand structure of which could conceivably either cause overrepresentation of DNA fragments originating from it in sequencing libraries or it could be non-specifically bound by antibodies during the immunoprecipitation process. To distinguish between these possibilities, we carried out the same analysis on DNase, FAIRE and MNase data. As these assays do not involve an immunoprecipitation step, they are a proper control for sequencing artifacts. We did not observe significant localized read enrichment in these datasets (Figure 7), suggesting that the observed read enrichment over the D-loop is not due to sequencing biases or overrepresentation of D-loop fragments in ChIP libraries. Similarly, we did not observe enrichment in the matched sonicated input ChIP-seq control datasets. However, a number of mock-immunoprecipitation (IgG) control datasets did exhibit high z-scores (up to >50 in K562 cells) and closely matched the signal profile over the D-loop of ChIP-seq datasets (Figure 8B). We also examined the forward and reverse strand read distribution in the NCR (Figure 8). Site-specific transcription factor binding events display a characteristic asymmetry in the distribution of reads mapping to the forward and reverse strands, with reads on the forward strand showing a peak to the left of the binding site and reads on the reverse strand showing a peak to the right of it [39] (Figure 8C). Such read asymmetry was not observed in the D-loop region (average profile shown in Figure 8A, individual dataset profile shown in Figure 1).

These results suggest that while immunoprecipitation is necessary for high enrichment over the D-loop, the enrichment might not be mediated by the proteins targeted by the primary antibody. This does not explain why a large number of factors show little enrichment over the D-loop (Figures 4, 5 and 6) and why some factors show enrichment that is much higher than that observed in K562 IgG controls, with z-scores of up to 300 (compared to a maximum of 50 for the most highly enriched IgG controls). Still, given the lack of clear hallmarks of site-specific occupancy, and the IgG control results, enrichment over the D-loop has to be provisionally considered to be primarily the result of an experimental artifact, even if it cannot be ruled that at least in some cases it is the result of real biochemical association with nuclear transcriptional regulators.

**Figure 9:**
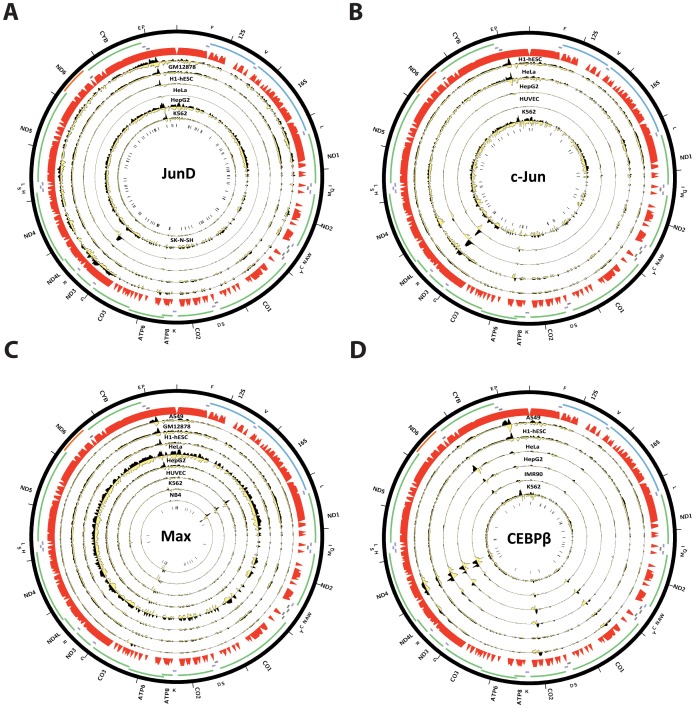
Human transcription factors with canonical ChIP-seq peaks (displaying the typical strand asymmetry in read distribution around the putative binding site) outside of the D-loop. Reads mapping to the forward strand are represented in black, reads mapping to the reverse strand are represented in yellow. The unique mappability track for the mitochondrial genome is shown in red in the outside track (see Methods for details). Protein-coding, rRNA and tRNA genes are shown as colored bars. The innermost circle shows the motif occurrences in the mitochondrial genome for each factor as black vertical bars. (A) JunD (B) c-Jun; (C) Max; (D) CEBP*β*. The reads per million (RPM) tracks are shown, scaled to the maximum signal level (for both strands) for each dataset. Plots were generated using Circos version 0.60 [41].

**Figure 10:**
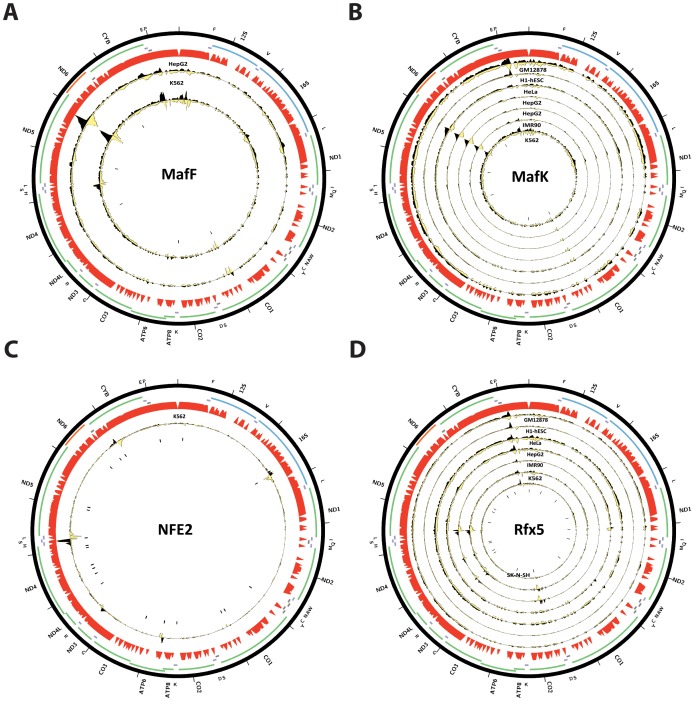
Human transcription factors with canonical ChIP-seq peaks (displaying the typical strand asymmetry in read distribution around the putative binding site) outside of the D-loop. Reads mapping to the forward strand are represented in black, reads mapping to the reverse strand are represented in yellow. The unique mappability track for the mitochondrial genome is shown in red in the outside track (see Methods for details). Protein-coding, rRNA and tRNA genes are shown as colored bars. The innermost circle shows the motif occurrences in the mitochondrial genome for each factor as black vertical bars. (A) MafF; (B) MafK (note that MafK has been assayed using two different antibodies in HepG2, both of which are shown); (C) NFE2; (D) Rfx5. The reads per million (RPM) tracks are shown, scaled to the maximum signal level (for both strands) for each dataset. Plots were generated using Circos version 0.60 [41].

In contrast to the widespread, but likely artifactual, read enrichment over the D-loop, we observed strong enrichment, exhibiting the canonical characteristics of a ChIP-seq peak over a true transcription factor binding site, in other regions of the human mitochondrial genome for eight of the examined transcription factors using a minimum z-score threshold of 20: CEBP*β*, c-Jun, JunD, MafF, MafK, Max, NFE2 and Rfx5. Figures 9 and 10 show the forward and reverse strand read distribution for representative replicates of each factor in each assayed cell line, as well as the occurrences of the corresponding explanatory motifs (identified from the top 500 ChIP-seq peaks in the nuclear genome, see Methods for details). The putative binding sites outside of the D-loop are characterized by an asymmetric forward and reverse strand read distribution, and in most cases, the presence of the explanatory motif in a position consistent with binding by the factor. We identified multiple binding sites for CEBP*β*: a strong site of enrichment around the 5′ end of the CYB gene, what seems to be two closely clustered sites in the ND4 gene, a weaker site in the ND4L gene, and two other regions of enrichment over CO2 and CO1 (Figure 9D). A single very strong binding site over the ND3 gene was observed for c-Jun, as well as two weaker sites, one coinciding with the ND4 CEBP*β* sites and one near the 5′ end of ATP6 (Figure 9B); the strong ND3 site was also observed for JunD in HepG2 cells. Max exhibited two putative binding sites: one in the middle of the 16S rRNA gene, containing a cluster of Max motifs, and another one around the 5′ end of CO3, which also contains a cluster of Max motifs but is in a region of poor mappability. A common and very strong MafK and MafF binding site is present near the 3′ end of ND5, though it does not contain the common explanatory motif for both factors (Figure 10AB). Several putative binding sites were identified for NFE2: one close to the CEBP*β* site in the 5′end of CYB, one over the tRNA cluster between ND4 and ND5, one in the 5′ end of ATP6 and one in the 16S rRNA gene (Figure 10C). Finally, two putative binding sites ar observed for Rfx5, at the 5′ end of ND5 and in the middle of CO2 (Figure 10D). Intriguingly, these binding events are not always present in all cell lines. For example, CEBP*β* binding around CYB was absent in K562, A549 and H1-hESC cells, while the MafK ND5 binding site was absent in GM18278 and H1-hESC cells, but present in the other cell lines for which data is available.

### Nuclear transcription factor occupancy to the mitochondrial genome in model organisms

**Figure 11:**
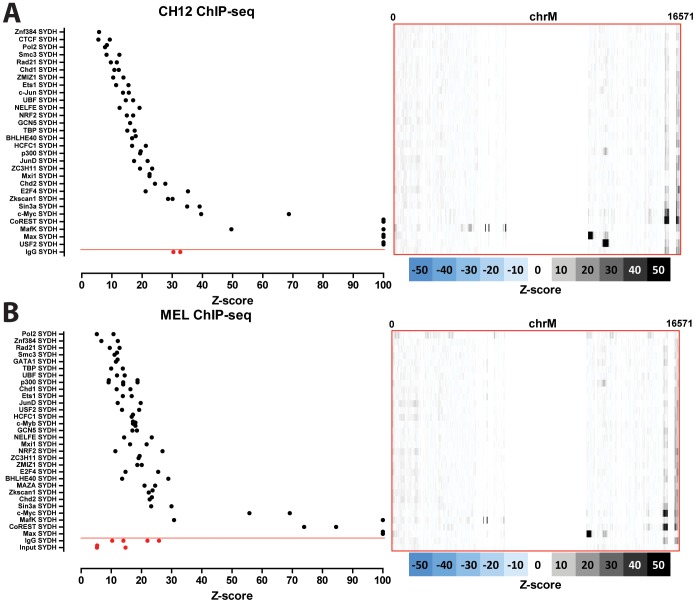
Signal distribution over the mitochondrial genome in mouse ChIP-seq datasets. Shown is the maximum z-score for each individual replicate for each cell line (left) and the z-score profile along the mitochondrial chromosome for the replicate with the highest z-score (right). Control datasets are shown in red on the bottom, below the red horizontal line. (A) CH12 cells; (B) MEL cells.

**Figure 12:**
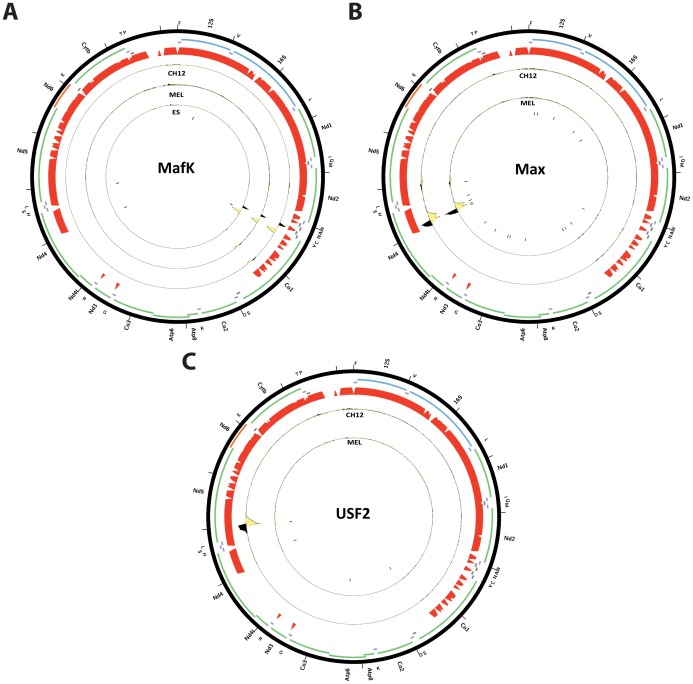
Mouse transcription factors with canonical ChIP-seq peaks (displaying the typical strand asymmetry in read distribution around the putative binding site) outside of the D-loop. Reads mapping to the forward strand are represented in black, reads mapping to the reverse strand are represented in yellow. The unique mappability track for the mitochondrial genome is shown in red in the outside track (see Methods for details). Protein-coding, rRNA and tRNA genes are shown as colored bars. The innermost circle shows the motif occurrences in the mitochondrial genome for each factor as black vertical bars. (A) MafK (note that the putative binding site is found in a region that is not completely mappable, thus the read profiles loses the canonical shape but the strand asymmetry is nevertheless apparent and a motif is present); (B) Max; (C) USF2. The reads per million (RPM) tracks are shown, scaled to the maximum signal level (for both strands) for each dataset. Plots were generated using Circos version 0.60 [41].

We carried out the same analysis as described above on mouse and *C. elegans* ChIP-seq datasets. Figure 11 shows the distribution of read density z-scores in mouse CH12 and MEL cells. Similarly to the human data, we observe widespread but probably artifactual read enrichment over the D-loop. In addition to that, we saw that three transcription factors (Max, MafK, and USF2) also exhibit strong enrichment elsewhere in the mitochondrial genome (Figure 12). We observe a single MafK binding site, containing the explanatory motif and situated over the tRNA cluster between the ND2 and CO1 genes (Figure 12A). Max displayed a strong binding site (possibly a cluster of closely spaced binding sites) in the ND4 gene, and a weaker binding site near the 5′ end of ND5; both sites contained the explanatory motif (Figure 12B). Finally, a single site, also containing the explanatory motif for the factor and situated near the ND5 Max site, was present in CH12 USF2 datasets (but not in MEL cells) (Figure 12C). MafK and Max were also assayed in human cells, and, as discussed above, putative mitochondrial sites were identified there for both, though not at obviously orthologous to those found in the mouse data positions in the genome. We also analyzed available ChIP-seq data for the mouse orthologs of c-Jun and JunD, which in human cells exhibited putative mitochondrial binding sites. In contrast to observation in human, we did not detect strong sites for either protein in mouse.

**Figure 13:**
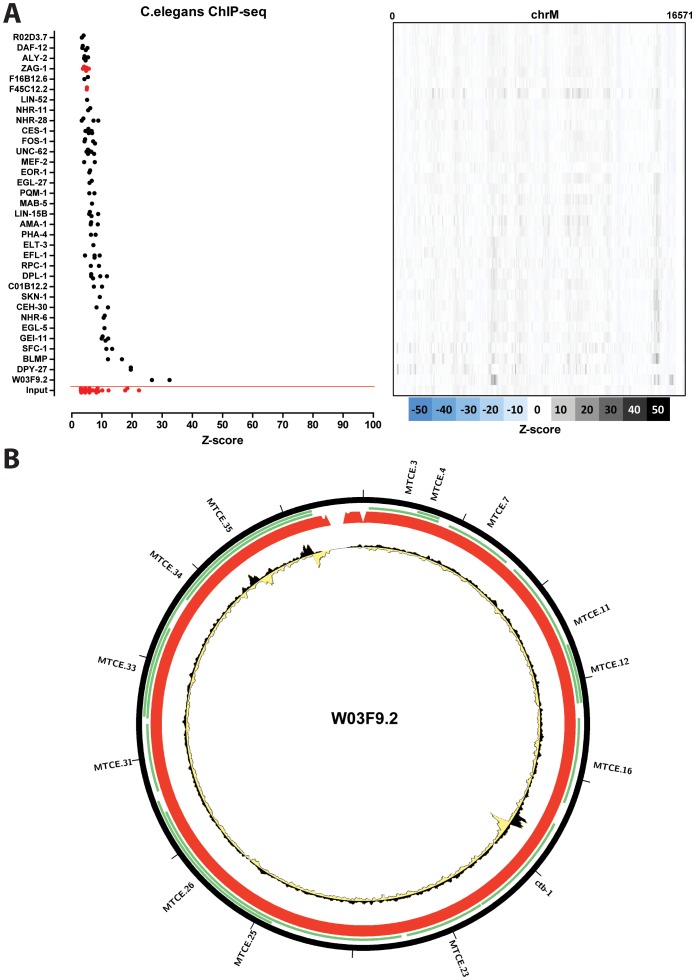
Signal distribution over the mitochondrial genome in *C.elegans* ChIP-seq datasets. (A) Shown is the maximum z-score for each individual replicate for each cell line (left) and the z-score profile along the mitochondrial chromosome for the replicate with the highest z-score (right). Control datasets are shown in red on the bottom, below the red horizontal line; (B) Forward and reverse strand read distribution over the *C.elegans* mitochondrial genome for W03F9.2 (“Young Adult” stage). Reads mapping to the forward strand are represented in black, reads mapping to the reverse strand are represented in yellow. The unique mappability track for the mitochondrial genome is shown in red in the outside track (see Methods for details). Plots generated using Circos version 0.60 [41].

Unlike the mouse and human datasets, most *C. elegans* ChIP-seq datasets did not show very strong enrichment over the mitochondrial genome (Figure 13A), with the exception of DPY-27 and W03F9.2. Of these, only W03F9.2 exhibited regions of enrichment with the characteristics of transcription factor binding sites (Figure 13B); however, very little is known about this protein and the significance of its binding to the mitochondrial genome is unclear.

### ChIP-seq signal is significantly stronger over mitochondrial occupancy sites than it is over nucleus sites

The occupancy observations reported above for human and mouse mitochondria do not formally rule out the possibility that there are unannotated NUMTs in the genomes of the cell lines in which binding is detected in our analysis and the observed binding is in fact nuclear. Such an explanation is superficially likely, given that binding to the mitochondrial genome was observed in some cell lines and not in others. However, closer examination reveals that this hypothesis would require different NUMTs in different cell lines as the cell lines that lack binding are not the same for all factors. For example, MafF and MafK binding is very prominent in K562 cells but CEBP*β* and c-Jun seem not to bind to mtDNA in those cells. While still possible, we consider the independent insertion of multiple partial NUMTs in different cell lines to be an unlikely explanation for the observed binding patterns.

**Figure 14:**
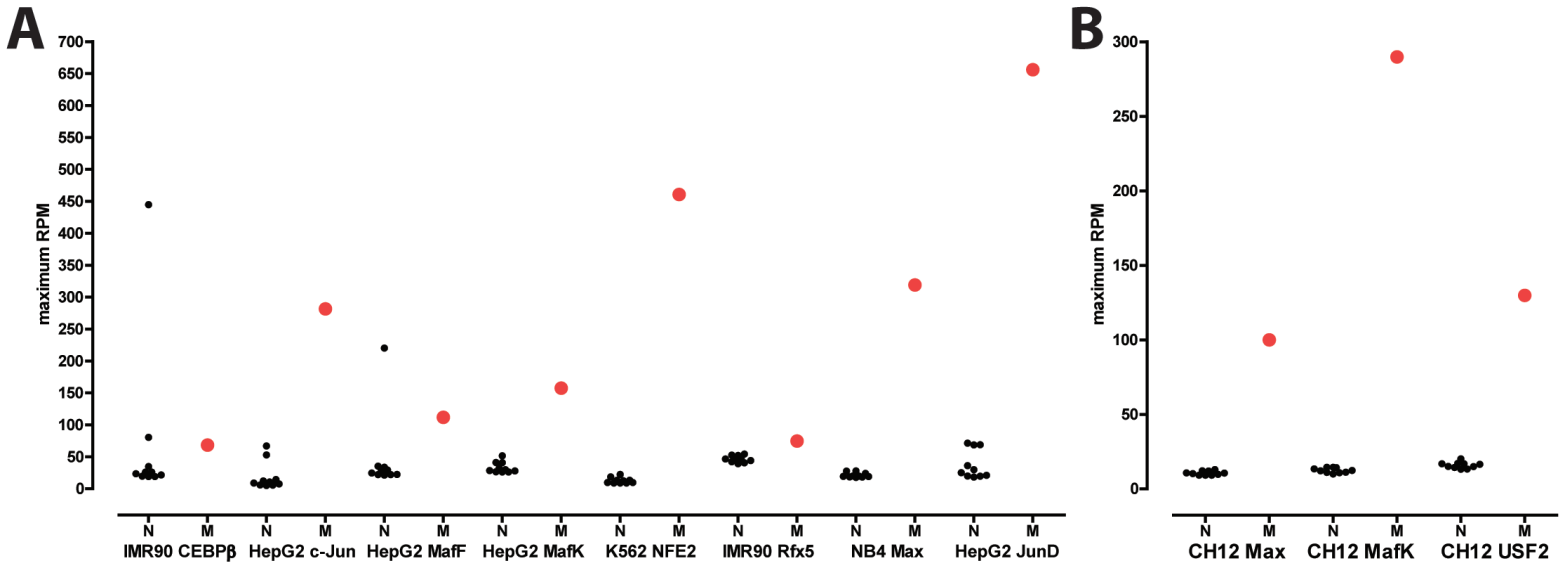
Mitochondrial ChIP-seq peaks are generally significantly stronger than nuclear peaks. Shown is the maximum signal (in RPM) for the top 10 nuclear peaks (“N”, smaller black dots), and the maximum signal intensity (also in RPM) in the mitochondrial genome (“M”, larger red dot) for representative ChIP-seq datasets for each factor. (A) Mouse datasets (B) Human datasets.

Each chromosome in the nuclear genome exists as only two copies in diploid cells, as compared to the hundreds of mitochondria, each of which may contain multiple copies of the mitochondrial genome [7], [64], and although cancer cells may exhibit various aneuploidies and copy number variants, the number of mtDNA copies is still expected to be much higher. Thus, higher read density over mitochondrial transcription factor binding sites than over nuclear ones is expected, assuming similar occupancy rates. We therefore used the strength of ChIP-seq signal over mitochondrial occupancy sites in order to test the hypothesis that they are in fact nuclear, and not mitochondrial in origin. We compared the peak height (in **R**eads **P**er **M**illion, RPM) of the top 10 nuclear peaks (peak calls generated by the ENCODE consortium were downloaded from the UCSC Genome Browser) with that of the putatively mitochondrial binding sites (Figure 14). We found that the mitochondrial binding sites are usually the strongest binding sites by a wide margin, or at least within the top three of all peaks. For example, while the strongest nuclear MafK peak in mouse CH12 cells has a peak height of 14.5 RPM, the mitochondrial binding site has a peak height of 290 RPM. These observations are difficult to explain as being the result of binding to unannotated NUMTs in the nuclear genome, but are entirely consistent with the hypothesis that these factors indeed bind to the large number of copies of the mitochondrial genome present in each cell.

### Evidence for localization of transcription factors to mitochondria

**Figure 15:**
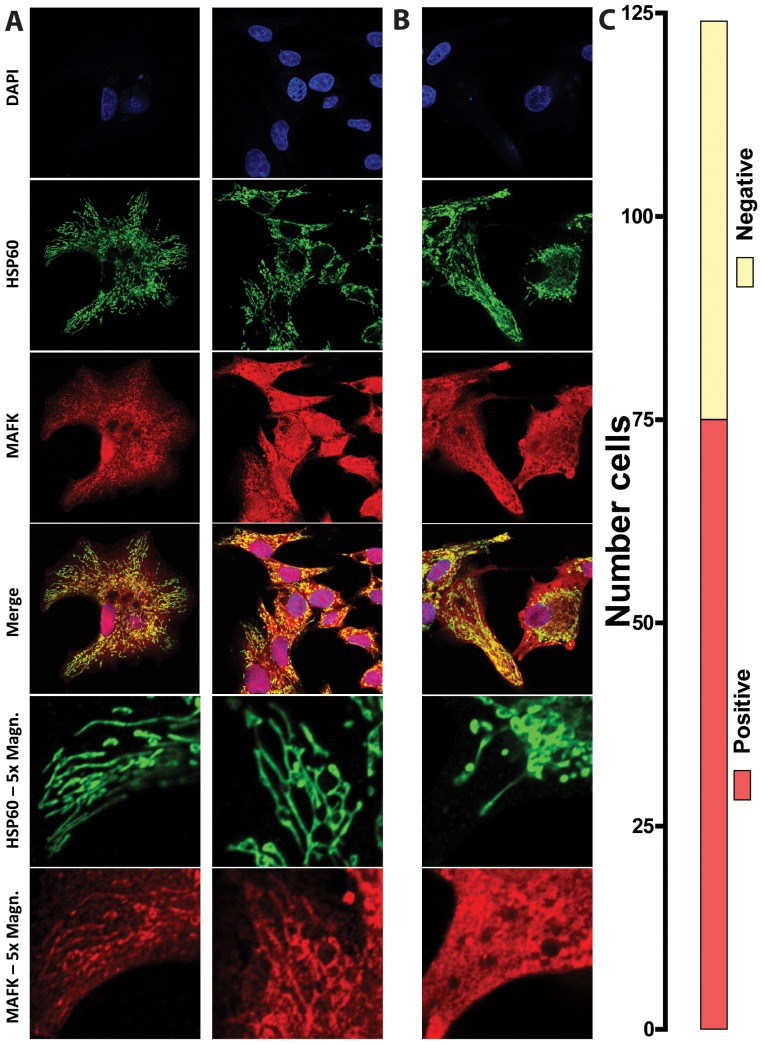
Localization of MafK to the mitochondria (A) Immunocytochemistry showing MafK localization in HepG2 cells. Mitochondria were identified by HSP60 staining. Shown are two representative images of cells showing that MAFK localizes strongly to the nucleus and mitochondria, and exhibits diffuse staining in the cytoplasm. In 60% of cells (C), there is colocalization of HSP60 with MAFK staining at an intensity higher than that of the surrounding cytoplasm. (B) An example of a cell exhibiting only nuclear and cytoplasmic MAFK localization.

If the observed binding sites in ChIP-seq data are the result of actual association of nuclear transcription factors with mtDNA, then these transcription factors should exhibit mitochondrial localization. We directly tested this by performing immunocytochemistry (ICC) for MafK in HepG2 cells (Figure 15). It is important to note that such an assay for localization to mitochondria is potentially difficult to interpret if binding is the result of only a few protein molecules entering mitochondria, which would not yield sufficient signal for interpretation via ICC. However, strikingly, we observe clear colocalization of MafK to mitochondira in 60% of cells (*n* = 124). These observations provide independent corroboration for the mtDNA binding events identified through ChIP-seq.

### No robust mitochondrial occupancy in ChIP-seq data for most previously reported mitochondrially targeted nuclear factors

**Figure 16:**
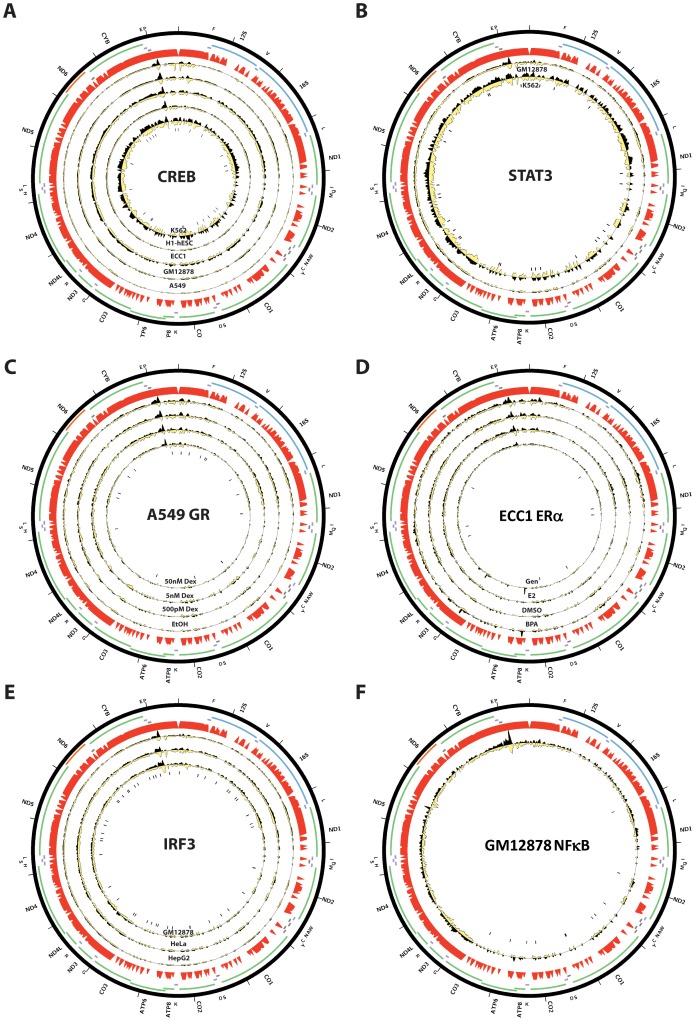
Distribution of reads over the human mitochondrial genome for factors previously reported to bind to mitochondria in ENCODE ChIP-seq data. Reads mapping to the forward strand are represented in black, reads mapping to the reverse strand are represented in yellow. The unique mappability track for the mitochondrial genome is shown in red in the outside track (see Methods for details). Protein-coding, rRNA and tRNA genes are shown as colored bars. The innermost circle shows the motif occurrences in the mitochondrial genome for each factor as black vertical bars. (A) CREB; (B) STAT3; (C) GR in A549 cells treated with different concentrations of dexamethasone (Dex) [60], [61]; (D) ER*α* in untreated (DMSO) ECC1 cells and ECC1 cells treated with bisphenol A (BPA), genistein (Gen) or 17*β*-estradiol (E2) [31]; (E) IRF3; (F) NF*κ*B in GM12878 cells treated with TNF*α* [37]. The reads per million (RPM) tracks are shown, scaled to the maximum signal level (for both strands) for each dataset. Plots were generated using Circos version 0.60 [41].

**Figure 17:**
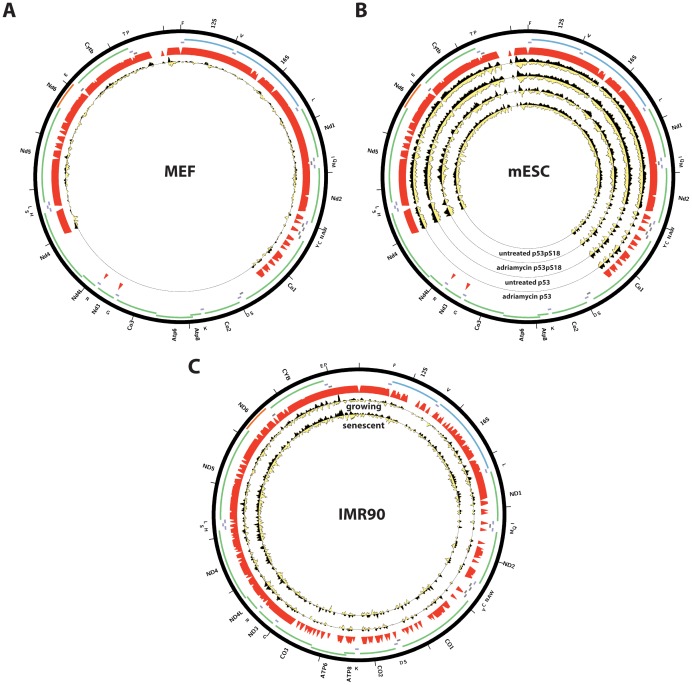
Distribution of reads over the human and mouse mitochondrial genome for p53 in publicly available ChIP-seq datasets. Reads mapping to the forward strand are represented in black, reads mapping to the reverse strand are represented in yellow. The unique mappability track for the mitochondrial genome is shown in red in the outside track (see Methods for details). Protein-coding, rRNA and tRNA genes are shown as colored bars. The innermost circle shows the motif occurrences in the mitochondrial genome for each factor as black vertical bars. (A) p53 in mouse embryionic fibroblasts (MEFs), data from [38], GSE46240. (B) p53 in mouse embryonic stem cells (mESC), data from [45], GSE26361; (C) p53 in human IMR90 cells, data from [2], GSE42728. The reads per million (RPM) tracks are shown, scaled to the maximum signal level (for both strands) for each dataset. Plots were generated using Circos version 0.60 [41].

**Figure 18:**
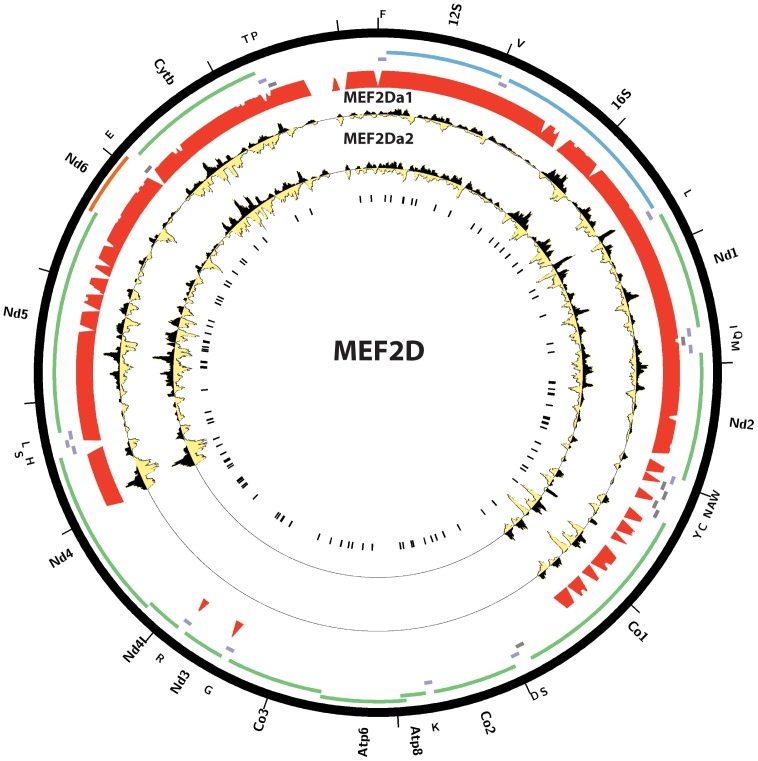
Distribution of reads over the mouse mitochondrial genome for MEF2D isoforms MEF2Da1 and MEF2Da2 in C2C12 myoblasts. Reads mapping to the forward strand are represented in black, reads mapping to the reverse strand are represented in yellow. The unique mappability track for the mitochondrial genome is shown in red in the outside track (see Methods for details). Protein-coding, rRNA and tRNA genes are shown as colored bars. The innermost circle shows the MEF2D motif occurrences in the mitochondrial genome as black vertical bars. Data was obtained from [65], GSE43223. Plots were generated using Circos version 0.60 [41].

We note that none of the factors previously reported to be localized to mitochondria and to bind to mtDNA was retrieved by our analysis, even though CREB, GR, ER*α*, IRF3, NF*κ*B, STAT1, STAT5A and STAT3 were assayed by the ENCODE Consortium. This failure could be attributed to the use of too stringent a z-score threshold when selecting datasets with significant enrichment. We therefore examined available ChIP-seq data against these factors more carefully (Figure 16, Figure S1). We also performed the same analysis on published mouse and human p53 ChIP-seq data [2], [38], [45] (Figure 17). Again, we did not observe any major sites of enrichment outside of the D-loop. For these factors, the D-loop region exhibits the same putatively artifactual pattern discussed previously. And for STAT3 and p53, even the enrichment over the D-loop was low. The one factor for which binding to mtDNA is confirmed by ChIP-seq is MEF2D, data for two of the isoforms of which in mouse C2C12 myoblasts was recently published [65] (Figure 18). It exhibits a very complex binding pattern over large portions of the mouse mitochondrial genome, which is not straightforward to interpet, but nevertheless a number of locations exhibit strand asymmetry and contain the MEF2 sequence recognition motif. Notably, most of these are outside the ND6 gene.

It is at present not clear how to interpret these discrepancies. It is not surprising that some of these factors do not exhibit binding to mtDNA, as they were reported to play a role in mitochondrial biology through mechanisms other than regulating gene expression (for example, IRF3 and STAT3). However, this is not the case for all of them. One possibility is that many prior studies reporting physical association of transcription factors with the D-loop suffered from the same artifactual read enrichment over that region that we observe, but this would not have been noticeable using the methods of the time. This would not be surprising, as it is only apparent that D-loop enrichment is likely to be artifactual when the high spatial resolution of ChIP-seq is combined with the joint analysis of input and mock immunoprecipitation controls. However, the mitochondrial localization of these factors has been carefully documented in a number of cases [8], [11], [17]. Another possiblity is that binding to mtDNA only occurs under certain physiological conditions and the factors were assayed using ChIP-seq only in cellular states not matching those. Further analysis of ChIP-seq data collected over a wide range of conditions should help resolve these issues.

## Discussion

We report here the first large-scale characterization of the association of nuclear transcription factors along the entire mitochondrial genome by utilizing the vast ChIP-seq data resource made publicly available by the ENCODE and modENCODE consortia. We find two classes of signal enrichment events, neither of which is detected in high-throughput sequencing datasets that do not involve immunoprecipitation and therefore they are not due to sequencing biases. First, the majority of factors for which we detect strong read enrichment over the mitochondrial genome display high ChIP-seq signal only over the D-loop non-coding region in both human and mouse datasets. However, these signals do not have the characteristics of sequence specific occupancy and are present in a number of mock-immunoprecipitation control datasets. They are thus best explained as experimental artifacts, although it remains possible that they represent real non-canonical association with the D-loop for some factors. Second, for a subset of factors, specific ChIP-seq peaks are observed outside of the D-loop, and these display the additional hallmark characteristics of sequence specific occupancy.

Nuclear transcription factors previously reported to localize to mitochondria either did not exhibit significant enrichment in the available ChIP-seq datasets or, when they did, it was over the D-loop region with similar non-specific read distribution shape as other factors. In contrast, applying conservative thresholds we found eight human and three mouse transcription factors (two in common between the two species) that strongly occupy sites outside of the D-loop. They display the strand asymmetry pattern around the putative binding site that typifies true nuclear ChIP-seq peaks. Even more convincing is the fact that the explanatory motif for the factor is usually found under the observed enrichment peaks, further suggesting that they correspond to true in vivo biochemical events.

There are three main explanations for our observations. First, it is possible that despite our considerable bioinformatic precautions the observed binding events are in fact nuclear, originating from NUMTs present in the genomes of the cell lines assayed, but absent from the reference genome sequence. We believe that this is very unlikely. An experimental argument against unknown NUMTs comes from the strength of the ChIP-seq signal we see in the mitochondrial genome. These signals are much higher than even the strongest peaks in the nuclear genome for the same factor in the same dataset. This is expected for true mitochondrial genome binding because of the presence of many copies of the mitochondrial genome per cell, in contrast to the presence of only two copies of the nuclear genome. Second, it is possible that mitochondria are sometimes lysed in vivo, with mitochondrial DNA spilling into the cytoplasm where transcription factors could then bind. This cannot be ruled out based on the ChIP data alone but we consider it unlikely, as this would need to happen with a sufficient frequency to explain the remarkable strength of mitochondrial occupancy sites. The third and most plausible interpretation is that these nuclear transcription factors indeed translocate to the mitochondria and interact with the genome, as has been observed for the D-loop in some previous studies for other factors. Indeed, immunocytochemistry experiments in our study confirm the presence of MafK in mitochondria in a majority of HepG2 cells.

Several major questions are raised by our results. First, it is not clear how these nuclear transcription factors are targeted to the mitochondria. Mitochondrial proteins are typically imported into the mitochondrial matrix through the TIM/TOM protein translocator complex, and are targeted to the organelle by a mitochondrial localization sequence, which is cleaved upon import. We scanned both human and mouse versions of our factors for mitochondrial target sequences (MTS) with both Mitoprot [15] and TargetP [21] (using default settings), but we were unable to identify significant matches using either. This seems to be a common feature of nuclear transcription factors previously found to localize to mitochondria, most of which lack import sequences and are instead imported through other means [11], [73]. Posttranslational modifications may be important for import, as has been demonstrated for STAT3 in TNF-induced necroptosis [68].

Second, it is unclear why the same factor binds detectably to the mitochondrial genome in some cell types but not in others. It is certainly possible that different splice isoforms or post-translationally modified proteins are present in different cell types, with only some capable of being imported into mitochondria, or that import into mitochondria only happens under certain physiological conditions only met in some cell lines.

Third, the question of the biochemical reality of transcription factor binding at the D-loop remains open. Previous studies understandably focused on the D-loop, given its well-appreciated importance in regulating mitochondrial transcription. As a consequence, the literature supporting a role for some nuclear factors in mitochondria suggests that they do so through binding to the D-loop. Our analysis of ChIP-seq data, which was carried out in an agnostic manner, revealed that dozens of transcription factors – many more than had been studied locally at the D-loop alone – also show high level of enrichment over the D-loop. However, the observed enrichment has characteristics suggesting that these signals are mainly due to experimental artifacts. In support of this judgment, the explanatory motifs for most of these factors were generally not found under the area of strongest enrichment in the D-loop. Therefore a conservative interpretation is that enrichment over the D-loop is an artifact in most cases.

Finally, and most importantly, the functional significance of factor occupancy observed by ChIP-seq remains unknown. It is entirely possible that it represents biochemical noise, with transcription factors entering the mitochondria because they have the right biochemical properties necessary to be imported, then binding to mtDNA but with little functional consequence. Alternatively, nuclear transcription factors may in fact be playing a regulatory role in mtDNA. It is difficult to imagine the exact mechanisms through which they might be acting, aside from interactions with the regulatory D-loop. While we do observe pairs of related factor such as c-Jun and JunD, and MafK and MafF binding to the same sites, binding events are overall widely dispersed over the mitochondrial genome and are found outside of the known regulatory regions. Plausible regulatory relationships are therefore not obvious and our results suggest that biological noise should be the working null hypothesis explaining the data. The functional regulatory role of these nuclear transcription factors in mitochondria is a very exciting possibility but it will have to be demonstrated in subsequent studies. Direct functional tests are the golden standard for establishing regulatory relationships, using gain and loss of function experiments and genetic manipulation of putative regulatory sites. The latter is at present not possible for mitochondria while the former are difficult to interpret in the case of the role of nuclear transcription factors in mitochondrial gene regulation, as it is not easy to separate the direct effects of binding to mtDNA from the indirect effects of transcriptional changes in the nucleus. Thus, it may be some time before definitive answers to these questions are obtained. In the meantime, larger compendia of transcription factor ChIP-seq data such as those expected to be generated by the next phase of the ENCODE project will be a primary source of further insight by providing binding data for additional nuclear transcription factors that will clarify allowed or preferred occupancy patterns across the mitochondrial genome.

## Materials and Methods

Except for where indicated otherwise, all analysis was carried out using custom-written python scripts.

### Sequencing read alignment

Raw sequencing reads were downloaded from the UCSC genome browser for ENCODE and mouseENCODE [54] data, and from ftp://ftp.modencode.org for modENCODE data [30], [50] (data current as of February 2012). ChIP-seq data for p53 was obtained rom GEO series GSE26361 [45], GSE46240 [38] and GSE42728 [2]. Reads were aligned using Bowtie [42], version 0.12.7. Human data was mapped against either the female or the male set of human chromosomes (excluding the Y chromosome and/or all random chromosomes and haplotypes) depending on the sex of the cell line (where the sex was known, otherwise the Y chromosome was included), genome version hg19. Mouse data was mapped against the mm9 version of the mouse genome. modENCODE *D. melanogaster* data was mapped against the dm3 version of the fly genome. modENCODE data for *C. elegans* was mapped against the ce10 version of the worm genome. Reads were mapped with the following settings: “-v 2 -k 2 -m 1 -t –best –strata”, which allow for two mismatches relative to the reference, however for all downstream analysis only reads mapping uniquely and with zero mismatches were considered, to eliminate any possible mapping artifacts.

### Mappability track generation

Mappability was assessed as follows. Sequences of length *N* bases were generated starting at each position in the mitochondrial genome. The resulting set of “reads” was then mapped against the same bowtie index used for mapping real data. Positions covered by *N* reads were considered fully mappable. In this case, *N* = 36 as this is the read length for most of the sequencing data analyzed in this study.

### Signal normalization of ChIP-seq data over the mitochondrial genome

Because the number of mitochondria per cell varies from one cell line/tissue to another, direct comparisons between datasets based on the absolute magnitude of the signal in RPM are not entirely valid. For this reason, we normalized the signal as follows. For each dataset, we fit a Gamma distribution over the RPM coverage scores for the bottom *F_b_* percentile of fully mappable position on the mitochondrial chromosome. The estimated parameters were then used to rescale the raw signal over all position to a z-score. This results in datasets with strong peaks receiving low z-scores over most of the mappable mitochondrial genome, and very high z-scores over the regions with highly localized enrichment. We used *F* = 0.8 for our analysis. As this procedure is sensitive to datasets with very low total read coverage over the mitochondrial genome, we restricted our analysis to datasets with at least 5000 uniquely mappable reads (and with no mismatches to the reference), i.e. ≥10*x* coverage. We used a z-score cutoff of 20 to select datasets with high enrichment over the mitochondrial genome, as it was the highest z-score observed in sonicated input samples

### Motif analysis

The peak calls for human and mouse ENCODE data available from the USCS Genome Browser were used to find de novo motifs for transcription factors from ChIP-seq data. The sequence around the peak summit (using a 50 bp radius) was retrieved for the top 500 called peaks for each factor in each cell line and motifs were called using the MEME program in the MEME SUITE, version 4.6.1 [4]. The MEME-defined position weight matrix was then used to scan the mitochondrial genome for motif matches following the approach described in [53].

### Cell growth and immunocytochemistry

HepG2 cells were grown following the standard ENCODE protocol (DMEM media, 4 mM L-glutamine, 4.5 g/L glucose, without sodium pyruvate, with 10% FBS (Invitrogen 10091-148) and penicillin-streptomycin). Cells were fixed in 10% formalin (Sigma-Aldrich HT501128-4L) for 10 min, permeabilized with 0.1% Triton X-100, and blocked in 5% FBS. Primary antibodies used were MafK (1∶100, Abcam, ab50322) and Hsp60 (1∶125, Santa Cruz, sc-1052). Secondary antibodies used were donkey anti-goat AF488 (Invitrogen A11055) and donkey anti-rabbit AF546 (Invitrogen A10040). Imaging on a Zeiss LSM 710 confocal microscope with PlanApochromat 63X/1.4 oil objective, and 0.7 µm optical sections were acquired.

## Supporting Information

Figure S1 **Distribution of reads over the human mitochondrial genome for STAT1 and STAT5A in ENCODE ChIP-seq data.** Reads mapping to the forward strand are represented in black, reads mapping to the reverse strand are represented in yellow. The unique mappability track for the mitochondrial genome is shown in red in the outside track (see Methods for details). Protein-coding, rRNA and tRNA genes are shown as colored bars. The innermost circle shows the motif occurrences in the mitochondrial genome for each factor as black vertical bars. (A) STAT1; (B) STAT5A; The reads per million (RPM) tracks are shown, scaled to the maximum signal level (for both strands) for each dataset. Plots were generated using Circos version 0.60 [41].
(PDF)
