# Supplementary material for: Evidence for Site-Specific Occupancy of the Mitochondrial Genome by Nuclear Transcription Factors
Source: PLoS One. 2014 Jan 20;9(1):e84713. doi: 10.1371/journal.pone.0084713 (PMC3896368; doi:10.1371/journal.pone.0084713)
Supplement: Figure S1 — Distribution of reads over the human mitochondrial genome for STAT1 and STAT5A in ENCODE ChIP-seq data. Reads mapping to the forward strand are represented in black, reads mapping to the reverse strand are represented in yellow. The unique mappability track for the mitochondrial genome is shown in red in the outside track (see Methods for details). Protein-coding, rRNA and tRNA genes are shown as colored bars. The innermost circle shows the motif occurrences in the mitochondrial genome for each factor as black vertical bars. (A) STAT1; (B) STAT5A; The reads per million (RPM) tracks are shown, scaled to the maximum signal level (for both strands) for each dataset. Plots were generated using Circos version 0.60 [41]. (PDF) [file pone.0084713.s001.pdf]

Supplementary Figures

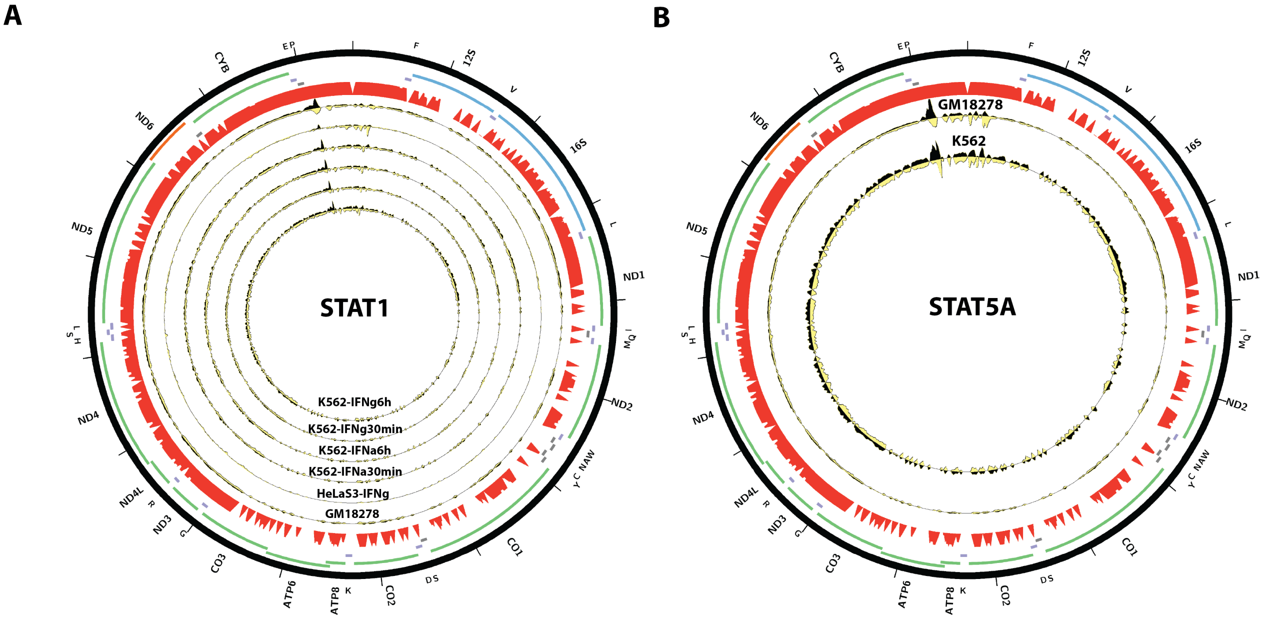

Supplementary Figure 1. Distribution of reads over the human mitochondrial genome for STAT1 and STAT5A in ENCODE ChIP-seq data. Reads mapping to the forward strand are represented in black, reads mapping to the reverse strand are represented in yellow. The unique mappability track for the mitochondrial genome is shown in red in the outside track (see Methods for details). Protein-coding, rRNA and tRNA genes are shown as colored bars. The innermost circle shows the motif occurrences in the mitochondrial genome for each factor as black vertical bars. (A) STAT1; (B) STAT5A; The reads per million (RPM) tracks are shown, scaled to the maximum signal level (for both strands) for each dataset. Plots were generated using Circos version 0.60 [**?**].
